# Molecular epidemiology of residual *Plasmodium vivax* transmission in a paediatric cohort in Solomon Islands

**DOI:** 10.1186/s12936-019-2727-9

**Published:** 2019-03-28

**Authors:** Yi Wan Quah, Andreea Waltmann, Stephan Karl, Michael T. White, Ventis Vahi, Andrew Darcy, Freda Pitakaka, Maxine Whittaker, Daniel J. Tisch, Alyssa Barry, Celine Barnadas, James Kazura, Ivo Mueller

**Affiliations:** 1 Population Health and Immunity Division, The Walter and Eliza Hall Institute of Medical Research, Parkville, VIC Australia; 2 Department of Medical Biology, The University of Melbourne, Parkville, VIC Australia; 3 Department of Parasites and Insect Vectors, Institut Pasteur, Paris, France; 4 National Health Training & Research Institute, Ministry of Health, Honiara, Solomon Islands; 5 College of Public Health, Medical and Veterinary Sciences, James Cook University, Townsville, QLD Australia; 6 Case Western Reserve University, Cleveland, OH USA

**Keywords:** *Plasmodium vivax*, Solomon Islands, Asymptomatic, Cohort, Heterogeneity

## Abstract

**Background:**

Following the scale-up of intervention efforts, malaria burden has decreased dramatically in Solomon Islands (SI). Submicroscopic and asymptomatic *Plasmodium vivax* infections are now the major challenge for malaria elimination in this country. Since children have higher risk of contracting malaria, this study investigated the dynamics of *Plasmodium* spp. infections among children including the associated risk factors of residual *P. vivax* burden.

**Methods:**

An observational cohort study was conducted among 860 children aged 0.5–12 years in Ngella (Central Islands Province, SI). Children were monitored by active and passive surveillances for *Plasmodium* spp. infections and illness. Parasites were detected by quantitative real-time PCR (qPCR) and genotyped. Comprehensive statistical analyses of *P. vivax* infection prevalence, molecular force of blood stage infection (_mol_FOB) and infection density were conducted.

**Results:**

*Plasmodium vivax* infections were common (overall prevalence: 11.9%), whereas *Plasmodium falciparum* infections were rare (0.3%) but persistent. Although children acquire an average of 1.1 genetically distinct *P. vivax* blood-stage infections per year, there was significant geographic heterogeneity in the risks of *P. vivax* infections across Ngella (prevalence: 1.2–47.4%, p < 0.01; _mol_FOB: 0.05–4.6/year, p < 0.01). Malaria incidence was low (IR: 0.05 episodes/year-at-risk). Age and measures of high exposure were the key risk factors for *P. vivax* infections and disease. Malaria incidence and infection density decreased with age, indicating significant acquisition of immunity. G6PD deficient children (10.8%) that did not receive primaquine treatment had a significantly higher prevalence (_a_OR: 1.77, p = 0.01) and increased risk of acquiring new bloodstage infections (_mol_FOB _a_IRR: 1.51, p = 0.03), underscoring the importance of anti-relapse treatment.

**Conclusion:**

Residual malaria transmission in Ngella exhibits strong heterogeneity and is characterized by a high proportion of submicroscopic and asymptomatic *P. vivax* infections, alongside sporadic *P. falciparum* infections. Implementing an appropriate primaquine treatment policy to prevent *P. vivax* relapses and specific targeting of control interventions to high risk areas will be required to accelerate ongoing control and elimination activities.

**Electronic supplementary material:**

The online version of this article (10.1186/s12936-019-2727-9) contains supplementary material, which is available to authorized users.

## Background

Over recent years, collaborative efforts among ministries of health in malaria-endemic countries, international communities and global funders have significantly decreased the transmission and clinical burden of malaria worldwide [1]. The decrease in transmission has resulted in *Plasmodium* spp. infections becoming more heterogeneous within countries, between regions, among villages and even within households [2–4]. The decline of clinical malaria has often been linked with an increase in low density and afebrile *Plasmodium* spp. infections [5–7]. These low-density infections are particularly problematic for malaria control and elimination programmes as they are not detected by routine surveillance and constitute a silent reservoir of infections [8, 9]. Asymptomatic infections may be chronic and untreated *Plasmodium falciparum* infections can persist for 6 months or more [10]. Meanwhile, asymptomatic *Plasmodium vivax* relapses may account for 80% of all *P. vivax* blood-stage infections [11].

In many malaria-affected countries outside sub-Saharan Africa, where both *P. falciparum* and *P. vivax* coexist, a shift in species predominance from *P. falciparum* to *P. vivax* has been observed [6, 12]. Given its strong preference to invade reticulocytes [13] and the more rapid acquisition of clinical immunity to *P. vivax* [14], low-density, asymptomatic infections tend to constitute a higher proportion of *P. vivax* infections as compared to *P. falciparum* infections. Conventional malaria surveillance approaches focused on monitoring clinical cases [15–18] are thus poorly suited to identify and delineate pockets of residual *P. vivax* transmission [19].

Following substantial progress made in malaria control, the Solomon Islands are aiming for malaria elimination by 2030 [20]. National statistics reported a tenfold decrease in malaria incidence over the last two decades from 442/1000 population in 1992 to 40.5/1000 population in 2015 [21]. In addition, *Plasmodium* spp. transmission is highly heterogeneous across provinces [22, 23], ranging from high transmission in Makira Province (80.6/1000 population in 2013) to low transmission in Isabel (5.8/1000 population in 2013), and no transmission in Rennell and Bellona Province [21]. Though impressive, these reductions in malaria burden may be fragile and in the context of reduced funding for malaria control and inadequate access to services reported malaria cases escalated to 81.0/1000 in 2016 [24, 25]. A national household survey in 2015 reported that while 86% of households in the SI own at least one long-lasting insecticide-treated net (LLIN), LLIN usage was reported to only be 57% [21]. In addition, the prolonged use of insecticides and LLINs in Solomon Islands has resulted in a tendency for Anopheline mosquitoes to bite more frequently outdoors during the early evening instead of biting indoors and later at night [26]. A more in-depth understanding of the nature and risk factors for residual malaria transmission is thus essential for improving the effectiveness of Solomon Islands’ malaria control and elimination programmes.

In the two provinces where pilot programmes for malaria elimination were conducted (Isabel and Temotu), an increase in the proportion of malaria caused by *P. vivax* infection were observed as the overall malaria incidence decreased. Furthermore, a higher proportion of low-density and asymptomatic infections, as well as increased spatial clustering of residual malaria transmission risk were observed [22, 27–29]. Less is known about how ongoing control has changed malaria epidemiology in the higher transmission areas of central Solomon Islands. National statistics (based on light microscopic diagnosis) in 2012 reported that 63% of malaria cases in Central Islands Province were caused by *P. falciparum* while the remaining 37% of malaria cases were due to *P. vivax* infections [21, 30, 31]. However, *P. vivax* prevalence by qPCR was considerably higher than that of *P. falciparum* (13% vs. 0.14%, respectively) in a cross-sectional survey of 3501 local residents in Ngella, Central Islands Province [23]. Findings from this survey also identified high rates of submicroscopic and asymptomatic *Plasmodium* spp. infections, and a higher risk of infections among children as compared to adults.

Since cross-sectional surveys only measure *Plasmodium* spp. infection at a single time point, it is difficult to understand how risk factors identified in the cross-sectional survey may be related to the seasonal patterns of *Plasmodium* spp. infections in Ngella. From a single-time point study, it is also not possible to determine incidence of *Plasmodium* spp. infection and illness. Therefore, an in-depth investigation on the burden of asymptomatic and submicroscopic *Plasmodium* spp. infections among children (6 months to 12 years of age) in Ngella was conducted as part of an 11-month longitudinal, observational cohort study. In order to describe the longitudinal dynamics of *Plasmodium* spp. infections and disease, 860 children from villages located in North Coast and Bay regions (previously identified with higher *Plasmodium* spp. transmission rate [23]) were followed and the incidence of *P. vivax* infections and its associating risk factors were studied.

## Methods

### Study site

**Fig. 1:**
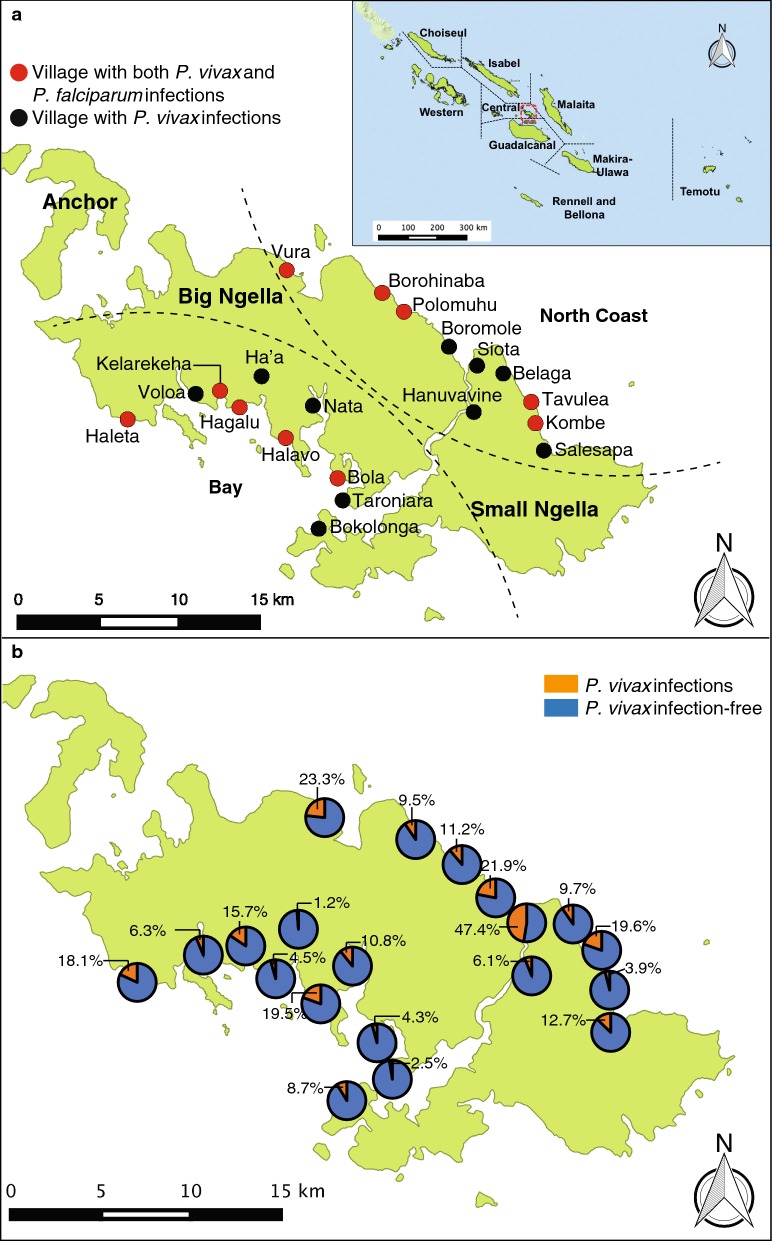
Ngella study sites and the distribution of *Plasmodium* spp. infections. **a** Participants were recruited from 20 villages in Ngella, Central Islands Province. Based on qPCR detection, *P. vivax* infections were identified in all villages while *P. falciparum* were detected in 10 villages (red dot). **b** Prevalence of *P. vivax* infections (qPCR detection) in Ngella villages (Map created using QGIS 2.10.1)

This study was conducted in Ngella, Central Islands Province of Solomon Islands. Ngella is located between Malaita (approximately 50 miles north east) and Guadalcanal provinces (approximately 27 miles south and hosts the capital city, Honiara). These provinces are connected to Ngella by ferry service and private boats. Their malaria incidences were shown to be moderate to high in 2013 (Honiara: 69.9/1000 population; Guadalcanal: 64.0/1000 population; Malaita: 35.9/1000 population; Central Islands: 55.5/1000 population) [21, 31]. Further details of the study site are described elsewhere [23]. A total of 20 villages in Ngella were selected for this study (Fig. 1) based on: (i) higher prevalence of *P. vivax* infections as noted previously among 10 villages in Ngella [23], (ii) consultation with Ministry of Health officials, (iii) easy accessibility during high tides and stormy weather throughout the year, as well as (iv) number of children in each village.

### Field study design

The study was conducted from May 2013 until April 2014 among children aged 6 months to 12 years old. A schematic overview of the cohort is presented in Additional file 1. After obtaining written informed consent from a parent or legal guardian, children were clinically examined (including weight, signs of anaemia, symptoms of sickness, permanent disability and chronic illness), and screened for glucose-6-phosphate dehydrogenase (G6PD) deficiency using the BinaxNOW G6PD (Alere Inc., USA) test kit. To assure appropriate test performance, these tests were stored at 4 °C and conducted on top of an ice bucket, in a well-aerated room (< 30 °C). Demographic information (including age, date of birth, sex, residency status, village of residency), LLIN usage, and past treatment of malaria were recorded. Children that fit the inclusion criteria (local healthy individual aged between 6 months and 12 years old with a normal weight that has given consent and completed all enrolment procedures) were invited to participate in the cohort study. Children with a chronic illness or permanent disability, living in a remote area and children that were not permanent residents were excluded. All eligible and consenting children were enrolled.

Participants were then actively followed for a period of 11 months at 4-weekly intervals, apart from a longer period (up to 6 weeks) between the 7th and 8th visit due to the Christmas holiday season. During each active case detection (ACD) visit, information on LLIN usage the night before by mother and child was recorded. The reported LLIN usage by the participants and their mothers was very similar and thus was combined and averaged the LLIN usage data from both mother and child during cohort. Tympanic temperature and antimalarial treatment history (from each child’s personal health book) were also recorded.

Any child presenting with fever (temperature ≥ 38 °C) or history of fever (recorded up to 2 weeks prior to examination) was clinically assessed by a health worker. If malaria was suspected, a rapid diagnostic test (RDT) (CareStart, Access Bio, USA) was performed. Treatment for malaria was given in accordance with the national malaria treatment guidelines: artemether–lumefantrine (Coartem^®^) treatment for *P. falciparum* infections; Coartem^®^ and 14 days of primaquine (PQ; 0.25 mg/kg, only in G6PD normal individual) for *P. vivax* infections. Guidelines require G6PD testing prior to PQ treatment. Haemoglobin (Hb) was measured if a child had signs of anaemia (conjunctiva, hand pallor, oedema, shortness of breath or feeling dizzy). Children were treated according to diagnosis and/or, when appropriate, referred to Tulagi Hospital. Participants with frequent absenteeism (less than 6 ACD visit attendances) were excluded from further analysis.

A capillary blood sample (approximately 250 μL) was collected in EDTA-Microtainer (Becton–Dickinson, NJ, USA) tubes from all participants. At the collection site, 50 μL of blood were immediately transferred into 250 μL RNAProtect (Qiagen, Germany) RNA stabilization reagent, and stored in an ice container (at approximately 4 °C) before transportation to the centralized field laboratory. Upon arrival at the centralized field laboratory (within 12 h of collection), samples in RNA stabilization reagent were frozen immediately at − 20 °C. The remaining blood samples were centrifuged at 300×*g* for 10 min to separate into cellular and plasma fractions, prior to storage at − 20 °C. Blood smears for light microscopy were prepared at ACD1, ACD8 and ACD11, and examined for parasites by experienced microscopists at the regional malaria laboratory. Information from blood smears positive for *Plasmodium* spp. was used to identify infected children during the survey by the local health workers for anti-malarial treatment. Hb levels were measured at ACD1, ACD6 and ACD11. At the last visit (ACD11), information on participant household indoor residual spraying (IRS) status in the previous 12 months was collected.

A passive case detection (PCD) system was maintained throughout the study at the local health facility levels (Tulagi hospital, local rural health centres and aid posts). Participants identified by PCD were clinically assessed and examined for malaria by RDT. Clinical diagnosis was recorded, and capillary blood samples were collected onto filter paper (Whatman 3MM, Maidstone, UK). Treatment was given to children as per diagnosis of the attending physician.

### Molecular characterization of *Plasmodium* spp. parasites

All collected blood cell pellets (frozen state) and filter paper blood spots (room temperature) were transported to the Walter & Eliza Hall Institute in Melbourne, Australia. Total genomic DNA was isolated from all blood cellular fractions and filter paper blood spots using FavorPrep 96-Well Genomic DNA Extraction Kits (Favorgen Biotech Corporation, Taiwan). All DNA extracts were screened for *P. falciparum* and *P. vivax* parasites using a duplex *P. falciparum*/*P. vivax* Taqman qPCR targeting the 18s gene [32]. Parasite densities by qPCR are expressed as 18s gene copy numbers/μL.

Samples positive for parasites were genotyped using molecular markers *msp1*F3, MS2 and MS16 for *P. vivax* infections, while *msp2*, TA81 and Polyα were used for *P. falciparum* infections [33, 34]. The genotyped samples were submitted for capillary electrophoresis, followed by fragment analysis using GeneMapper software (Applied Biosystems, USA), as described previously [33].

From the genotyping data, the multiplicity of infection (MOI) and molecular force of blood-stage infection (_mol_FOB) were determined [35, 36]. Briefly, _mol_FOB is the observed number of new blood-stage infections, as identified by individual *Plasmodium* spp. genotypes, divided by the time-at-risk (i.e. the incidence of new blood-stage infections). An observed infection, as identified by a specific genotype, was considered to be ‘new’ if the same genotype (of any molecular marker) had not been seen in the two previous active or passive case detection visits. As such, _mol_FOB can be determined for each study participant or as a sum over the entire study population or subpopulations.

### Alpha (α)-thalassaemia genotyping

All children were genotyped for the two most common forms of α-thalassaemia: 3.7 kbp (α^−3.7^) and the 4.2 kbp deletions (α^−4.2^). A multiplex PCR reaction described elsewhere [37] was used and the genotypes were identified by sizing patterns on agarose gel.

### Statistical analyses

Field data were double entered using a REDCap electronic data capture system. All statistical analyses were conducted using STATA (v.12.1, StataCorp) and were focused mainly on *P. vivax* infections as there were too few *P. falciparum* infections to conduct meaningful statistical analysis, except for the analysis of incidence of clinical episodes, where both *P. vivax* and *P. falciparum* data were used. The analyses performed were similar to a previously described paediatric cohort study in Papua New Guinea [38]. Three types of analyses were conducted for *P. vivax* infections: (1) prevalence of infection, (2) incidence of new infection, and (3) infection density. All three analyses were done using a generalized estimating equation (GEE) population-averaged model with the semi-robust Huber/White/sandwich estimator of variance. This approach was taken due to the irregular data structure that included missing data.

The prevalence of *P. vivax* infection (1) throughout the period of the study was analysed using logistic regression based on the XTLOGIT command in STATA. Only the ACD visits data were included in the analysis of prevalence as the study was estimating the prevalence at each visit. The incidence of new *P. vivax* clones acquired during cohort (2) was assessed using a negative binomial regression approach based on the XTNBREG command in STATA [35]. New *P. vivax* clones acquired during PCD, as determined using the technique described above were binned into the following ACD visit interval for consistency. *Plasmodium vivax* log-transformed infection densities (3) were analysed by linear regression method using the XTREG command in STATA. Incidences of clinical malaria (both *P. falciparum* and *P. vivax* infections) were analysed using the Andersen and Gill model of multiple time failure survival [39]. Backward elimination using the Wald’s Chi square test for individual variables was used to select the best fitting models in all statistical analyses. Associations between risk predictors from models were assessed using Chi square test, Kruskal–Wallis test or one-way analysis of variance (ANOVA), depending on the variable type.

## Results

### Study demographics

**Fig. 2:**
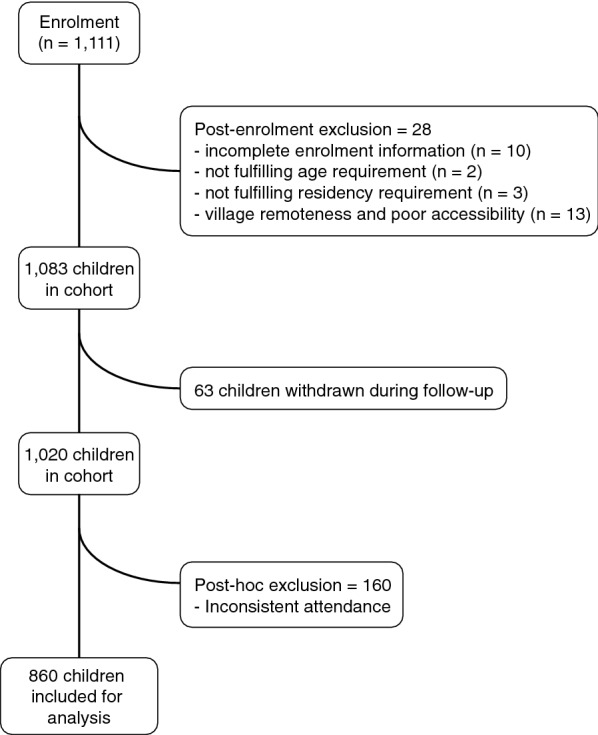
Inclusion flowchart of children in the Solomon Islands observational cohort

A total of 1111 children aged 6 months to 12 years old from 20 villages in Ngella (Fig. 1) were enrolled during May 2013. Of these, data from 860 children was kept for analysis (Fig. 2). Among the 251 excluded children, 28 children were excluded post-enrolment (refer to Fig. 2 for details), 63 children withdrew from the study; and 160 children were excluded post hoc due to inconsistency in follow-up attendance. The excluded participants (Additional file 2) were similar in gender and age to those included, but due to problems with accessibility of villages a significantly higher number of children residing in the North Coast region were excluded as compared to the Bay region (p-value < 0.001). The 860 children included in the final analyses had a minimum of 6 ACD attendances (Additional file 3).

The demographic characteristics of the 860 included children are listed in Additional file 4. These children came from 469 households, had a median age at enrolment of 5.6 years (IQR: 3.0–8.4 years), and were 48.4% female and 51.6% male. Almost all of children (99%) were local Ngella residents. The number of children enrolled from the 20 villages varied, ranging from 14 (Nata) to 78 (Polomuhu). Each region (Bay and North Coast) was approximately equally represented. Mean usage of LLIN was high at 88%, and 89% of households were protected by IRS.

During enrolment, 7.2% of children reported feeling ill. Based on health book records, there were 65.0% children with at least one previous antimalarial treatment record (PATR), and 35.4% of children reported having received antimalarial treatment less than 1 year prior to the start this study. Although past parasite species had not been recorded for these previous episodes, it was observed that Coartem^®^ was most commonly given (75.7%), while PQ treatment was less frequent (20.0%).

The average Hb measurements from ACD1 (June 2013), ACD6 (November 2013) and ACD11 (April 2014) showed that 49.8% of children were anaemic (Hb < 110 g/L). There were 10.8% of children diagnosed as G6PD-deficient, of which 63.4% were male. Meanwhile, 34.8% of children had at least one mutated α-thalassaemia gene (89.3% children with heterozygous polymorphisms and 10.7% children with homozygous polymorphisms; refer to Additional file 4), with the − α^3.7^ being the dominant deletion mutation (72.8%).

### Burden of *Plasmodium* spp. infections

**Fig. 3:**
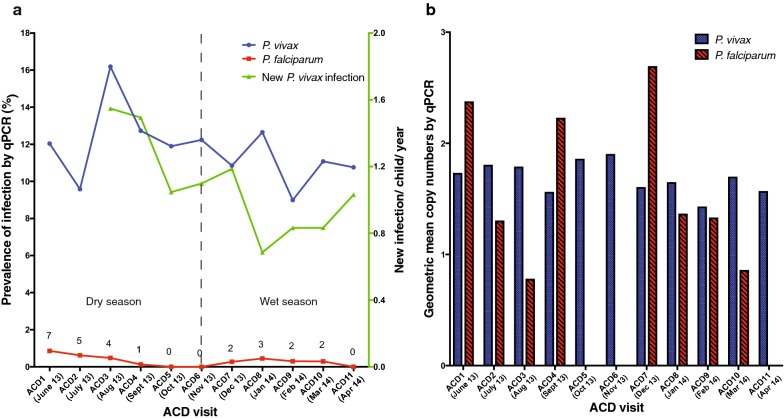
*Plasmodium* spp. infections at ACD visits. **a** Prevalence of qPCR-detected *P. falciparum* (red curve) and *P. vivax* (blue curve) infections by monthly ACD follow-up visits (left y-axis). New *P. vivax* infections (green curve, right y-axis) was identified through new genotypes observed in consecutive follow-up blood samples from participants during cohort. **b** Infection density of 18S rRNA gene of *P. falciparum* and *P. vivax* by qPCR detection

A total of 8329 blood samples were collected and analysed. Based on qPCR screening, 989 *P. vivax* infections (average prevalence: 11.9%; 976 observations from ACD visits) and 27 *P. falciparum* infections (overall prevalence: 0.32%; 26 observations were from ACD visits) were identified, six of which were mixed infections with *P. vivax*. *Plasmodium falciparum* infections were detected in children from ten villages (Fig. 1). Four of 22 children were positive for *P. falciparum* more than once. These observations indicate that the level of local *P. falciparum* transmission in Ngella is very low. In contrast, ongoing transmission of *P. vivax* infection was observed in Ngella without marked seasonality (Fig. 3). *Plasmodium vivax* infections showed pronounced clustering among children. Only 294 children (34.2%) had one or more positive *P. vivax* blood-stage infections during follow-up (Additional file 5). This is significantly fewer than predicted if infections were randomly distributed among children (expected 68.3%, p < 0.001). Among those with detected infections, 181 children (21.1%) had two or more *P. vivax* infections during the follow-up (expected 31.9%, p < 0.001) (Additional file 5). Although there was a higher prevalence of G6PD deficiency among male children, the prevalence of *P. vivax* infections was similar among G6PD deficient individuals of both genders (13.6% vs. 8.8% by microscopy or RDT detection, 37.3% vs. 35.3% by qPCR detection; Additional file 4).

### Genetic diversity, MOI and _mol_FOB

**Fig. 4:**
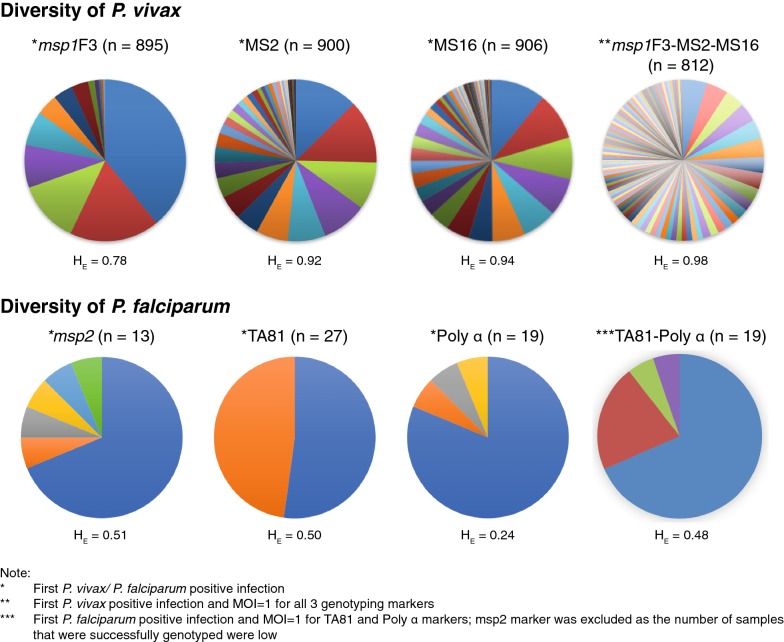
Contrasting diversity of *P. vivax* and *P. falciparum* in Ngella. Allelic frequencies of *P. vivax* markers: *msp1*F3, MS2, MS16 and the combined *msp1*F3-MS2-MS16 haplotypes and *P. falciparum* markers: *msp2*, TA81, Polyα and the combined TA81-Polyα haplotypes. The number of successfully genotyped *Plasmodium* spp. isolates or combined haplotypes are shown above each respective pie chart. Each estimated expected heterozygosity (H_E_) are given below the figure

Of the *P*. *vivax* positive samples, 90% were successfully genotyped for *msp1*F3 alleles, 91% for both MS2 and MS16 alleles. Meanwhile, only 16 of 27 (59%) of *P. falciparum* infected samples were successfully characterized for *msp2* genotypes, but 96% and 70% of the *P. falciparum* samples were successfully genotyped by TA81 and Polyα, respectively (Additional file 6). Based on the calculated virtual heterozygosity (H_E_) of the first genotyped *Plasmodium* spp. infection of each infected participant, the *P. vivax* alleles (H_E_ of *msp1*F3 = 0.78; H_E_ of MS2 = 0.92; H_E_ of MS16 = 0.94; and H_E_ of 3 markers combined = 0.98) were found to be more diverse than the *P. falciparum* alleles (H_E_ of *msp2* = 0.51; H_E_ of TA81 = 0.50; H_E_ of Polyα = 0.24; and H_E_ of TA81 and Polyα markers combined = 0.48) (Fig. 4).

In *P. vivax* infections, the MOI ranged from 1 to 3, with 73% of infections exhibiting MOI = 1. The _mol_FOB was 0.62 (CI_95_ 0.53–0.72; range 0–14.47) new *P. vivax* infections per child per year-at-risk by *msp1*F3, 0.75 by MS2 (CI_95_ 0.64–0.86; range 0–9.95) and 0.72 by MS16 (CI_95_ 0.61–0.83; range 0–12.79). Combining all three molecular markers, the average _mol_FOB was 1.06 (CI_95_ 0.90–1.21, range 0–21.70).

Almost all identified *P. falciparum* infections exhibited MOI = 1, except for three with MOI = 2. A proportion of 69% of the genotyped *P. falciparum* clones shared the same 508 bp *msp2* FC27 subtype allele, which was also observed previously in the cross-sectional survey during the previous year [23]. For *P. falciparum*, the average _mol_FOB was 0.02 (CI_95_ 0.01–0.04; range 0–2.91).

### Asymptomatic and submicroscopic *Plasmodium* spp. infections

A total of 433 children had at least one febrile episode detected during the active follow-up (ACD) visits. Of these children, 73 were positive by qPCR (71 *P. vivax* and 4 *P. falciparum* including two mixed infections). As such, an estimated 7.2% of *P. vivax* and 14.8% of *P. falciparum* infections were associated with febrile symptoms. All febrile *P. falciparum* and mixed infections but only 46% of *P. vivax* infections fulfilled the definition of clinical malaria based on febrile assessment and positive parasite detection by RDT or microscopy [40].

Among the 2250 blood films examined during the first (ACD1—June 2013), mid-cohort (ACD8—January 2015) and last visits (ACD11—April 2014), only 78 (3.5%) blood films were parasite positive by microscopy (76 *P. vivax*, 4 *P. falciparum* including two mixed infections) (Additional file 7). Using these data, the proportion of asymptomatic and submicroscopic infections in these 3 follow-up visits were determined: 94% (247/264) of *P. vivax* and 90% (9/10) of *P. falciparum* infections were asymptomatic, while 71% (188/264) of *P. vivax* and 60% (6/10) of *P. falciparum* infections were submicroscopic (Additional file 7).

### Risk predictors for *P. vivax* infection

Risk factors for infection prevalence, molFOB and infection density are given in Table 1.

**Table 1:** Risk predictors of *P. vivax* (A) infection prevalence, (B) new infection, and (C) infection density by qPCR detection

	Overall *P. vivax* prevalence (%)	(A) Prevalence			(B) New *P. vivax* infection			(C) *P. vivax* density		
		OR^a^	aOR	95% CI	IRR^a^	aIRR	95% CI	Coeff.^a^	aCoeff.	95% CI
Age										
Linear	n/a	1.16*	1.18	[1.13, 1.23]	1.18*	1.21	[1.16, 1.25]	n/a		
Quadratic		n/a			n/a			− 0.01*	0.00	[− 0.01, − 0.002]
			p–value < 0.001			p-value < 0.001			p-value < 0.001	
Villages (for details refer to Additional file 10)	1.2–47.4	0.13–8.32	0.18–6.86	[0.02, 17.52]	0.07–4.96	0.11–6.18	[0.01, 12.42]	− 0.59–0.67	− 0.66–0.35	[− 0.95, 0.98]
			p-value < 0.001			p-value < 0.001			p-value < 0.001	
ACD follow-up timepoint (for details refer to Additional file 10)	9.0–16.2	0.72–1.39	0.69–1.46	[0.50, 1.86]	0.46–0.95	0.45–0.93	[0.32, 1.28]	− 0.25–0.14	− 0.47–0.15	[− 0.73, 0.38]
			p-value < 0.001			p-value < 0.001			p-value < 0.001	
LLIN usage (per 10% increase in use)	n/a	0.94*	0.89	[0.82, 0.97]	0.97	n/a		0.03	n/a	
			p-value = 0.006							
molFOB of *P. vivax*	n/a	n/a			n/a			0.02	0.02	[0.001, 0.04]
									p-value = 0.036	
Previous antimalarial record (1 year before only)	20.9	2.61*	2.03	[1.45, 2.83]	1.95*	1.52	[1.16, 1.98]	0.45*	0.24	[0.07, 0.41]
			p-value < 0.001			p-v`alue = 0.002			p-value = 0.005	
G6PD deficient	16.9	1.56*	1.77	[1.16, 2.70]	1.42	1.51	[1.05, 2.18]	−0.11	n/a	
			p-value = 0.008			p-value = 0.026				
α-Thalassaemia mutation^b^	14.2	1.56*	n/a		1.58*	1.42	[1.10, 1.84]	−0.06	n/a	
						p-value = 0.007				
Febrile	15.1	1.21	n/a		n/a			0.62*	0.51	[0.10, 0.92]
									p-value = 0.014	

Statistical analyses used were: (A) logistic regression method for prevalence analysis, (B) negative binomial regression method for incidences of new infection analysis, and (C) linear regression method for infection density analysis

*OR* odds ratio, *aOR* adjusted odds ratio, *95% CI* 95% confidence intervals for adjusted estimates, *IRR* incidence rate ratio, *aIRR* adjusted incidence rate ratio, *Coeff.* coefficient, *aCoeff.* adjusted coefficient

Data with asterisk (*) denotes significance (p-value < 0.05)

^a^Univariate data

^b^Children with at least one mutated α-thalassaemia gene

#### *Plasmodium vivax* infection prevalence

Prevalence of *P. vivax* was found to be significantly associated with increased age, G6PD deficiency, LLIN usage, previous antimalarial treatment record (PATR), village of residence and ACD visits (Table 1). *Plasmodium vivax* prevalence also varied over time (p = 0.002) but did not show a clear seasonal trend (Fig. 3a and Additional file 8). The risk of having a *P. vivax* infection increased linearly with age among children in Ngella (adjusted odds ratio, _a_OR: 1.18, CI_95_ [1.13–1.23], p < 0.001). The risk of *P. vivax* infection varied among villages (p < 0.001): Compared with Borohinaba, Siota was identified as having the highest *P. vivax* infections risk (_a_OR: 6.86, CI_95_ [2.68–17.5]) while Ha’a and Taroniara showed the lowest risk of infections (_a_OR: 0.18, CI_95_ [0.022–1.41] and _a_OR: 0.18, CI_95_ [0.050–0.62], respectively). The North Coast region of Ngella had a higher infection risk compared to the Bay region (Additional file 9). Despite this, heterogeneity of infection was observed; children living in Kombe (North Coast region) had a much lower risk of infection (_a_OR: 0.66, CI_95_ [0.28–1.59]) while children living in Halavo (Bay region) had a relatively high risk of infection (_a_OR: 3.15, CI_95_ [1.50–6.64]). These differences between villages were stable throughout the cohort study (Additional file 10).

With every 10% increased of the average LLIN usage, there was an 11% significant decrease in odds ratio, thus suggesting significant protective effect against *P. vivax* infections by LLIN (_a_OR: 0.89, CI_95_ [0.82–0.97], p = 0.006). Indoor residual spraying (IRS, based on data of 754 participants only) was associated with protection against *P. vivax* infections (OR: 0.42, CI_95_ [0.272–0.650], p < 0.001) in univariate (Additional file 9A) but not in multivariate analyses.

Children with PATR within 1 year of the start of the cohort study showed a higher risk of *P. vivax* infection (_a_OR: 2.03, CI_95_ [1.45–2.83], p < 0.001). Similarly, G6PD-deficient children were observed to have higher risk of being infected by *P. vivax* (_a_OR: 1.77, CI_95_ [1.16–2.70], p = 0.008). α-Thalassaemia mutation was found to increase (OR: 1.43, CI_95_ [1.07–1.93], p = 0.017) the risk of *P. vivax* infections) in univariate analyses only. There were no significant associations between being anaemic (Hb < 11 g/dL) and risk of *P. vivax* infections (Additional file 9A).

#### Acquisition of new *P. vivax* blood-stage infection

Acquisition of new, genetically distinct *P. vivax* blood-stage clones (i.e. _mol_FOB) was also strongly associated with age, villages, visit intervals, PATR, G6PD deficiency and α-thalassaemia mutation (Table 1b). As for prevalence, the risk of acquiring new *P. vivax* clones increased linearly with age (adjusted incidence rate ratio, _a_IRR: 1.21, CI_95_ [1.16–1.25], p < 0.001). Likewise, there was significant heterogeneity in risks among villages (p < 0.001). Risk of new *P. vivax* infections decreased substantially from third visit (reference visit) to last visit (_a_IRR: 0.61, CI_95_ [0.44–0.86]), with fluctuations between visit intervals (Fig. 3a). Children with PATR within 1 year preceding the start of the cohort study (_a_IRR: 1.52, CI_95_ [1.16–1.98], p = 0.002), those with G6PD deficiency (_a_IRR: 1.51, CI_95_ [1.05–2.18], p = 0.026) and/or α-thalassaemia mutations (_a_IRR: 1.42, CI_95_ [1.10–1.84], p = 0.007), were all more likely to acquire new *P. vivax* infections.

LLIN usage did not show any significant association univariately (Additional file 9B) or multivariately with risk of new *P. vivax* infections. Meanwhile, children living in a sprayed household (based on data of 754 participants only; IRR: 0.548, CI_95_ [0.37–0.82], p = 0.004) were protected from new *P. vivax* infections based on univariate analysis (Additional file 9B). Anaemic children (IRR: 0.696, CI_95_ [0.52–0.92], p = 0.012) were observed to have protection against new *P. vivax* infections based on univariate analysis (Additional file 9B). However after adjustment for age, G6PD deficiency, α-thalassaemia mutation, PATR, villages and visit intervals, household spraying and anaemic status were no longer significantly associated with incidence of new *P. vivax* infections.

#### *Plasmodium vivax* infection density

Among children with *P. vivax* infections detected during ACD visits (n = 291; Table 1c), age, PATR, _mol_FOB, febrile presentation, villages and study visit intervals were significantly associated with the geometric mean density of *P. vivax* infections. The *P. vivax* infection density decreased quadratically (_a_Coefficient: − 0.004, CI_95_ [− 0.006 to − 0.002], p < 0.001) with age and varied significantly among villages (p < 0.001). Contrary to infection risk, a seasonal pattern was observed for *P. vivax* infection density (Fig. 3b) with parasite densities significantly higher during the dry season (ACD1 to ACD5, June–October 2013) as compared to the wet season (ACD6 to ACD11, November 2013–April 2014). Overall, parasitemia decreased significantly (p < 0.001) with increasing time of follow-up.

Children presenting with febrile illness had *P. vivax* infections with higher parasitaemia (Coefficient: 0.51, CI_95_ [0.10–0.92], p = 0.014). Similarly, *P. vivax* infections among children with higher *P. vivax* exposure were also observed to have significantly higher parasite density (_a_Coefficient: 0.02, CI_95_ [0.001–0.04], p = 0.036; per unit increase in _mol_FOB). Likewise, *P. vivax* infected children with PATR within 1 year prior to the start of cohort had significantly higher parasite densities (_a_Coefficient: 0.24, CI_95_ [0.07–0.41], p = 0.005).

Reduction of parasitaemia was observed to be significantly associated with IRS univariately (Coefficient: − 0.29, CI_95_ [− 0.56 to − 0.02], p = 0.035; Additional file 9C) and multivariately (_a_Coefficient: 0.91, CI_95_ [− 1.37 to − 0.45], p < 0.001; Additional file 8), though this observation is based on only the 256 infected children with IRS information instead of all infected children (n = 291). G6PD deficiency, α-thalassaemia mutation, mean LLIN usage and anaemia were not correlated with *P. vivax* infection density.

### Malaria incidence

Due to unforeseen staffing and reporting problems, the quality of passive case detection was highly variable during follow-up. To overcome this issue, three distinct definitions of malaria episodes were used: (1) *confirmed malaria episode* (febrile and concurrent parasitaemia diagnosed by microscopy or RDT), (2) *presumptive malaria* (missing ACD morbidity and PCD records but documented antimalarial treatment in the child’s personal health book), and (3) *potential malaria* (febrile and positive detection of *Plasmodium* spp. by qPCR). Since the qPCR TaqMan detection method is a highly sensitive and more accurate as compared to RDT or light microscopy, all malaria species were corrected based on qPCR species typing to reduce the possibility of misinterpreted RDT results at field sites (13 misdiagnosed *P. vivax* infections as *P. falciparum* in the RDT results were identified). In Solomon Islands, only patients with RDT or light microscopy-confirmed malaria receive anti-malarial treatment. However, the infected species is not generally recorded in the patient’s health book. The combination of confirmed (Definition 1) and presumptive (Definition 2) malaria is thus most likely to reflect the true overall burden of clinical malaria episodes reliably.

There were 36 confirmed, 97 presumptive, and 39 potential malaria cases. *P. vivax* accounted for most of the clinical infections (94.6% in confirmed and 97.4% in potential malaria cases). The PATR (adjusted hazard ratio, _a_HR: 2.23, CI_95_ [1.45–3.42], p < 0.001) and the number of new infections acquired (_mol_FOB: 2.31 (cube root-transformed), CI_95_ [1.77–3.02], p < 0.001) were associated with increased risk of confirmed and presumptive malaria (i.e. definitions 1 and 2), while age (_a_HR: 0.89, CI_95_ [0.83–0.95], p = 0.001) decreased the risk. The same association of PATR and _mol_FOB with increased risk were also found for confirmed cases only (Definition 1) and all confirmed, presumptive and potential cases (Definition 1–3), whereas the decreased incidence with age was found only for the latter (Additional file 11).

### *Plasmodium falciparum* infections

Only 27 *P. falciparum* infections were observed during the cohort. The densities of these infections vary based on qPCR detection, ranging from 5 to 133,000 copy numbers/μL, while the median of parasite densities was 10.5 copy numbers/μL (IQR: 5–148 copy numbers/μL). Although the occurrence of *P. falciparum* was observed to be sporadic in Ngella, four individuals with repeated *P. falciparum* infections during follow-up visits were residing in the Bay region (two living in Halavo, and one each from Haleta and Bola). Of these four individuals, only one had a febrile episode once. Gametocyte detection by qPCR was not conducted but no *P. falciparum* gametocytes were detected during the three ACD visits’ microscopy examinations.

## Discussion

This study presents the first prospective cohort study focusing on *Plasmodium* spp. infection dynamics in Solomon Islands. As recognized in earlier cross-sectional studies in Ngella [23], and in the pilot elimination provinces in Temotu [27] and Isabel [22], most *Plasmodium* spp. infections detected from the cohort were submicroscopic and asymptomatic *P. vivax* infections. This is in line with the general trend of an increasing proportion of submicroscopic *Plasmodium* spp. infections as malaria transmission decreases [5, 7, 41]. *Plasmodium falciparum* infections which was previously thought to be nearly eliminated in Ngella [23] remained low in occurrence but persisted across Ngella sporadically.

The incidence of clinical malaria was low among cohort children and when species determination was possible, *P. vivax* accounted for most cases. This contrasts with the annual national malaria report by the National Vector-borne Disease Control Programme, Ministry of Health and Medical Sciences of Solomon Islands, which reported that approximately half of the malaria incidence in Central Islands was due to *P. falciparum* (49% and 47% of malaria cases in 2013 and 2014, respectively) [21]. Although information retrieval was attempted by checking for clinical malaria records in the health book of all children, it is likely that the true burden of malaria has been underestimated. It is also possible that *P. falciparum* episodes were more common among cases treated at the health facilities as compared to those diagnosed during active case detection. However, it has been previously recognized that the accuracy of clinical malaria case reporting in primary health care facilities in Solomon Islands is often relatively poor [42]. In addition, the results of this study are well in line with the changes observed at a remote region of Malaita, the neighbouring island to Ngella, where in parallel with a 91% reduction in all malaria cases, the proportion of clinical cases due to *P. vivax* increased from 33% in 2008 to 84% in 2013 [43]. While further studies will be required to determine the relative contribution of *P. falciparum* and *P. vivax* to the burden of clinical malaria in Ngella, data of this study make it clear that the very high burden of asymptomatic, low-density *P. vivax* infections is the key challenge to future malaria elimination in Ngella.

Although *P. vivax* infections were found throughout the study area, a very high degree of spatial heterogeneity in transmission was observed among the study villages. Prevalence varied up to 40-fold (range 1.2–47.4%) while the _mol_FOB varied up to 90-fold (range 0.05–4.57) between villages that were not more than 5–25 km apart. Such strong variation in malaria transmission levels at small geographical scales has been described before in both moderate to high transmission areas [44, 45] and are even more pronounced in low transmission settings [46, 47]. While the exact reasons for the persistence of these pockets of high transmission remain to be elucidated, the finding has important implications for current control and future elimination efforts in Solomon Islands. The high degree of heterogeneity will require spatial-targeting of malaria control and elimination programmes, where the high-risk villages (i.e. Siota) can be both a ‘sentinel site’ for surveillance as well as a target for enhanced control efforts, which includes active case detection and/or presumptive mass drug treatment [3, 48].

Although the burden of *P. vivax* infections varied during follow-up, seasonal patterns, while present, were modest. After a small drop in prevalence from ACD1 to ACD2, which was due to the fact that children with LM-positive infections detected at baseline (n = 52) received antimalarial treatments, prevalence and incidence of infections peaked in August (i.e. ACD 3) and then decreased during further follow-up. This coincided with a significant decrease in infection densities during the rainy season (ACD6 to ACD11; November–April), indicating that infection during the rainy season could be due to relapsing rather than newly acquired *P. vivax* infection. According to a previous environmental study in Northern Guadalcanal (west of Ngella), rainfall has a negative effect on *Anopheles farauti* larvae numbers as heavy rainfall flushes away the breeding soil ridges by the stream [49], thus explaining this somewhat surprising decrease in transmission levels during the rainy season. In addition to these seasonal effects, the overall declining infections could also be a result of the “healthy cohort” effect [50–52], as participation in an intensive cohort provides participants both with better access to healthcare, and makes their parents or guardians more aware of the disease.

Despite more than two-thirds of Anopheline mosquitoes in Solomon Islands bites outdoor and early (before 21.00 h) [26, 53], LLINs were still associated with a moderate protective effect against *P. vivax* infection prevalence among cohort children. Distribution of LLINs is undertaken every 3 years as the main vector control intervention in Solomon Islands and a 1.5 people/net ratio/population was reported for 2013 in Central Islands province [21, 54]. High LLIN ownership was also reported in a later nationwide survey (2015) with 85.9% of households owning at least one LLIN. However, only 56.8% people reported sleeping under the bed net [21]. The reasons for not using LLINs are complex but increasingly higher usage indicated that the awareness campaigns conducted along with the distribution of LLIN are working. Although a tendency towards a reduction was seen in the univariate analyses, LLIN use was not associated with significant reduction in incidence of new *P. vivax* blood-stage infections. It remains to be confirmed if this is a consistent effect. In Solomon Islands, IRS is considered to be a complementary intervention to LLINs [55] and only high-risk villages are selected for implementation with varying insecticide dosage [21]. Despite incomplete information on IRS among households, children living in sprayed households had significantly fewer *P. vivax* infections and lower parasite densities. This supports the continued use of IRS not merely as a complementary intervention in high risk areas but also in elimination areas where interruption of *Plasmodium* spp. transmission is the ultimate goal [56].

Despite relatively low levels of transmission, children in Ngella acquire substantial levels of clinical immunity to *P. vivax*. Although the prevalence of infections, the incidence of new infections, and thus exposure (_mol_FOB), all increase with age and exposure is a major risk predictor of clinical *P. vivax* malaria and parasite density, the incidence of clinical *P. vivax* episodes itself is not only very low but also decreases with age as does the mean density of *P. vivax* infections. These observed trends are signs of significant natural acquisition of clinical immunity against *P. vivax.* Similarly rapid acquisition of immunity to *P. vivax* was not only observed in a high transmission setting in PNG, where children start acquiring immunity to *P. vivax* in their 2nd year of life [38] and clinical *P. vivax* illness is virtually absent by age five [57], but also in lower transmission settings [14]. Although acquisition of immunity differs by endemicity settings and the exact rate of acquisition is unclear, the more rapid acquisition of immunity to *P. vivax* is largely due to the higher _mol_FOB [36, 58] driven by the combined burden of new and relapsing infections [11].

Having suffered an episode of malaria in the previous 12 months (previous anti-malarial treatment record or PATR) was found to be a strong risk predictor for infection prevalence, infection density, new infection, and malaria incidence. A higher risk of *P. vivax* infection in children with PATR suggests that treatment policy for *P. vivax* malaria in Solomon Island is sub-optimal. Although primaquine radical cure is part of the Solomon Islands’ standard treatment guidelines, it is often not prescribed due to the lack of G6PD testing and when prescribed, it is taken by the patient without supervision. A recent analysis of > 60,000 patients in Indonesian Papua found that unsupervised PQ was associated with only a minimal reduction in the risk of clinical *P. vivax* recurrences in the next 12 months [59]. As relapse infections can account for 80% of *P. vivax* blood-stage infections [11, 60], the high rate of recurrent *P. vivax* infections in children with PATR underlines that PQ treatment was either not given or not successful in removing hypnozoites from the liver, leading to future relapses. This effect is likely to be confounded by significant inter-individual heterogeneity in *P. vivax* infection risk as indicated by the high degree clustering of *P. vivax* infections among individuals. Having had a previous *P. vivax* infection is thus not only an indication that the child may carry further hypnozoites in its liver, but also indicates children with past exposures are significantly more likely to be exposed to new infectious mosquito bites in future. While _mol_FOB is a marker for current exposure to *P. vivax* infection, PATR is thus also a marker of (recent) past exposure.

The important contribution of relapses to the burden of *P. vivax* is further highlighted by the observation that G6PD deficient children had a higher prevalence of *P. vivax* infection and increased risk of acquiring new infection. This observation conflicts with previous reports that G6PD deficiency confers protection against severe *P. falciparum* malaria and clinical *P. vivax* infections and *P. vivax* parasitaemia [61–63]. While G6PD deficiency may protect against high *P. vivax* parasitaemia and thus clinical disease, the higher risk among G6PD deficient children is more likely due to the fact that these children represented a group that could not be treated with PQ, and therefore only received blood stage treatment. This again underlines the importance of providing efficient anti-relapse treatment and the need to find alternative treatment or prevention strategies for G6PD-deficient individuals.

Observation of low *P. falciparum* prevalence that are sporadic and largely asymptomatic occurring throughout the study areas and follow-up period is intriguing. Even more so as the National Vector-borne Disease Control Programme [21] reports that half of all clinical cases in Ngella are due to *P. falciparum* (see “Discussion” above). The observed *P. falciparum* in this cohort show low levels of genetic diversity and a large proportion (69%) carry the same *msp2* FC27 allele as the five *P. falciparum* infections detected in the previous year’s cross-sectional survey [23]. Likewise, there were low genetic diversity based on TA81 and Polyα characterization. Together, these observations suggest that endemic transmission of a near-clonal *P. falciparum* populations may persist in Ngella, similar to that previously observed in elimination province Temotu [64]. Further in-depth analyses of *P. falciparum* population genetics in Ngella and comparisons to neighbouring populations, as well as a detailed assessment of clinical isolates compared to asymptomatic infections will be required to better understand the nature of residual *P. falciparum* transmission in Ngella. In particular, it will be essential to understand what proportion and what infections are imported from neighbouring islands, which have much higher endemicity.

## Conclusions

This study has confirmed the presence of a significant burden of *P. vivax* infection and the sporadic occurrence of *P. falciparum* in Ngella, largely asymptomatic and sub-microscopic. The Solomon Islands malaria control programme currently relies almost entirely on vector control measures (such as LLINs and IRS) and management of clinical cases. While these approaches which have jointly achieved an overall 95% reduction in malaria cases in the last two decades remain effective, they are unlikely to achieve elimination of malaria transmission in Solomon Islands on their own. Alternative approaches that are better able to identify and target these silent reservoir *Plasmodium* spp. infections will be required. Higher sensitivity diagnosis tools such as loop-mediated isothermal amplification (LAMP) or PCR may be required to track these infections, and using active surveillance method rather than relying solely on passive surveillance method may be required to aid malaria elimination in Solomon Islands [65–67]. While active case finding and reactive case detection [48, 68, 69] may help with eliminating residual foci of *P. falciparum* transmission, further progress with eliminating *P. vivax* will crucially depend on attacking the hidden, hypnozoite reservoir [11]. A first priority would be the implementation of safe and efficacious PQ treatment (including G6PD testing) for all *P. vivax* infected individuals [70]. Further concerted efforts combining accurate identification of pockets of residual high transmission with focal mass drug administration may be required to detect and eliminate the reservoir of asymptomatic and submicroscopic *P. vivax* infections.

## Additional files

**Additional file 1: Figure S1.** Schematic overview of the paediatric cohort study in Ngella, Solomon Islands.

**Additional file 2: Table S1.** Characteristics of children that met inclusion criteria vs excluded children.

**Additional file 3: Table S2.** Retention of cohort participants and malaria prevalence during active case detection.

**Additional file 4: Table S3.** Detailed demographic characteristics of 860 children included in the cohort study.

**Additional file 5: Figure S3.** Frequency of *Plasmodium vivax* infections among cohort participants (n=860).

**Additional file 6: Figure S4.** *Plasmodium falciparum* genotypes variants that were successfully characterized by *msp2*, TA81 and Polyα markers.

**Additional file 7: Figure S5.** Asymptomatic and submicroscopic *Plasmodium* spp. infections during ACD1, ACD8 and ACD11 visits.

**Additional file 8: Table S5.** (i) Association between selected risk factors and *Plasmodium vivax* infection density by qPCR detection of the 256 participants with at least 1 *P. vivax* infection during cohort and household spraying data. (ii) Detailed estimates for village and visit interval effects for (A) *Plasmodium vivax* infection prevalence, (B) new *P. vivax* infection, and (C) *P. vivax* infection density by qPCR detection.

**Additional file 9: Table S4.** Univariate analysis of risk factors associated with (A) *Plasmodium viva*x infection, (B) new *P. vivax* infection and (C) *P. vivax* density by qPCR detection.

**Additional file 10: Figure S2.** Prevalence of *Plasmodium vivax* infection in villages during ACD visits.

**Additional file 11: Table S6.** Risk factors for incidence of clinical episodes: (A) Confirm malaria, presumptive malaria and potential malaria, (B) Confirm malaria.
