# Supplementary figures and images for: Molecular epidemiology of residual Plasmodium vivax transmission in a paediatric cohort in Solomon Islands
Source: Malar J. 2019 Mar 28;18:106. doi: 10.1186/s12936-019-2727-9 (PMC6437916; doi:10.1186/s12936-019-2727-9)

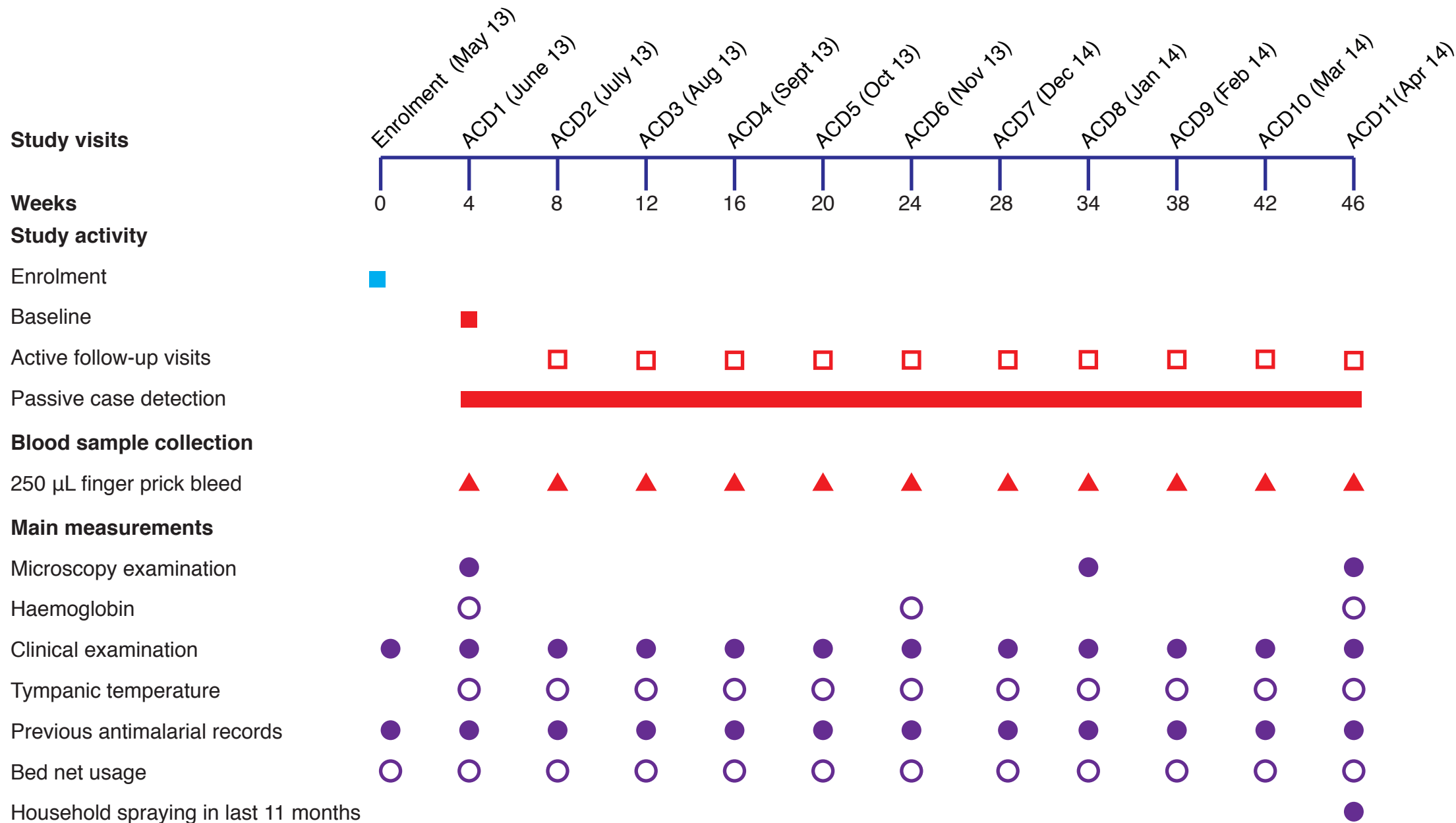

Supplement: Supplementary file 1 — Additional file 1: Figure S1. Schematic overview of the paediatric cohort study in Ngella, Solomon Islands. [file 12936_2019_2727_MOESM1_ESM.pdf]

Frequency of *P. vivax* infections among participants

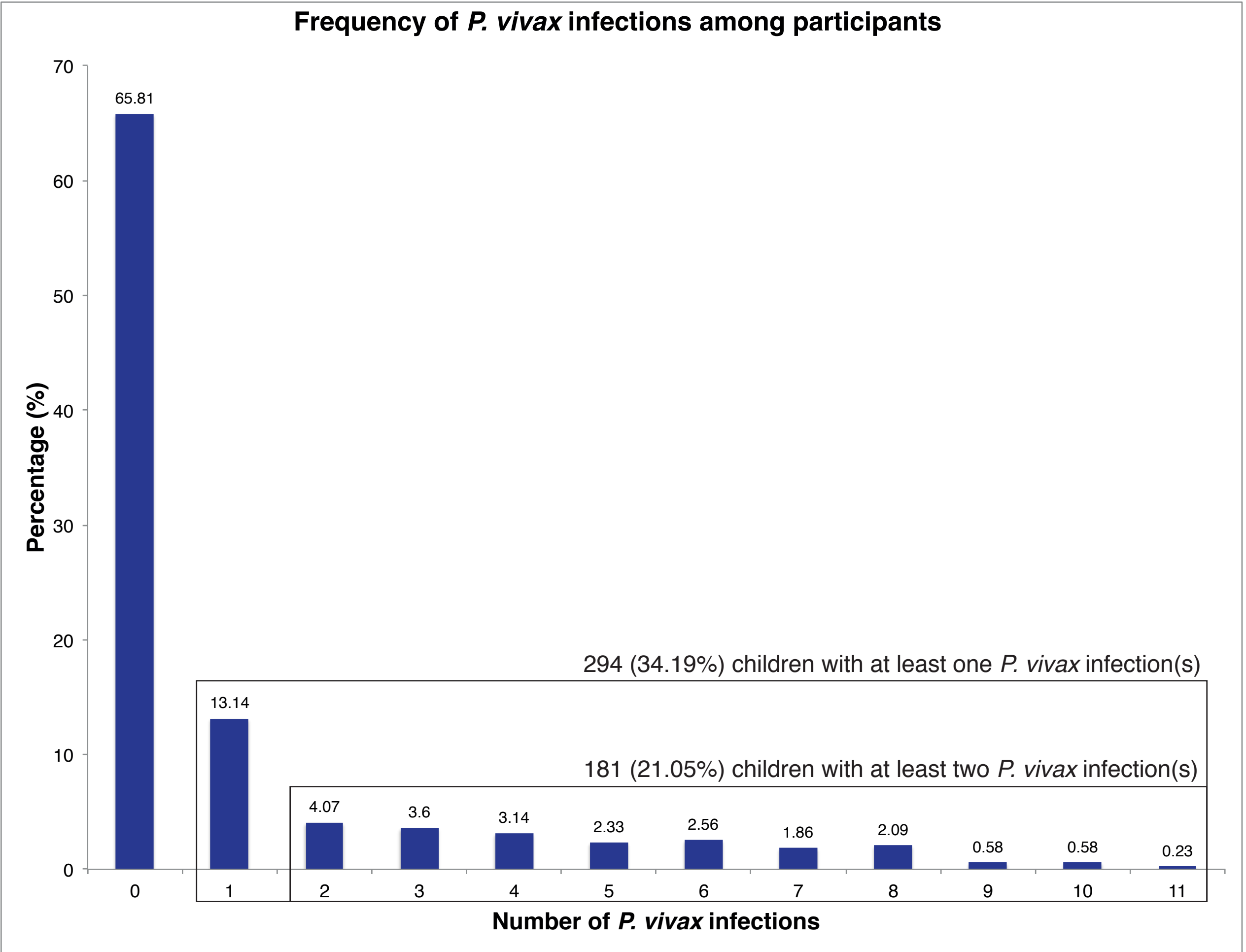

Supplement: Supplementary file 5 — Additional file 5: Figure S3. Frequency of Plasmodium vivax infections among cohort participants (n=860). [file 12936_2019_2727_MOESM5_ESM.pdf]

# Prevalence of *Plasmodium vivax* infection in villages

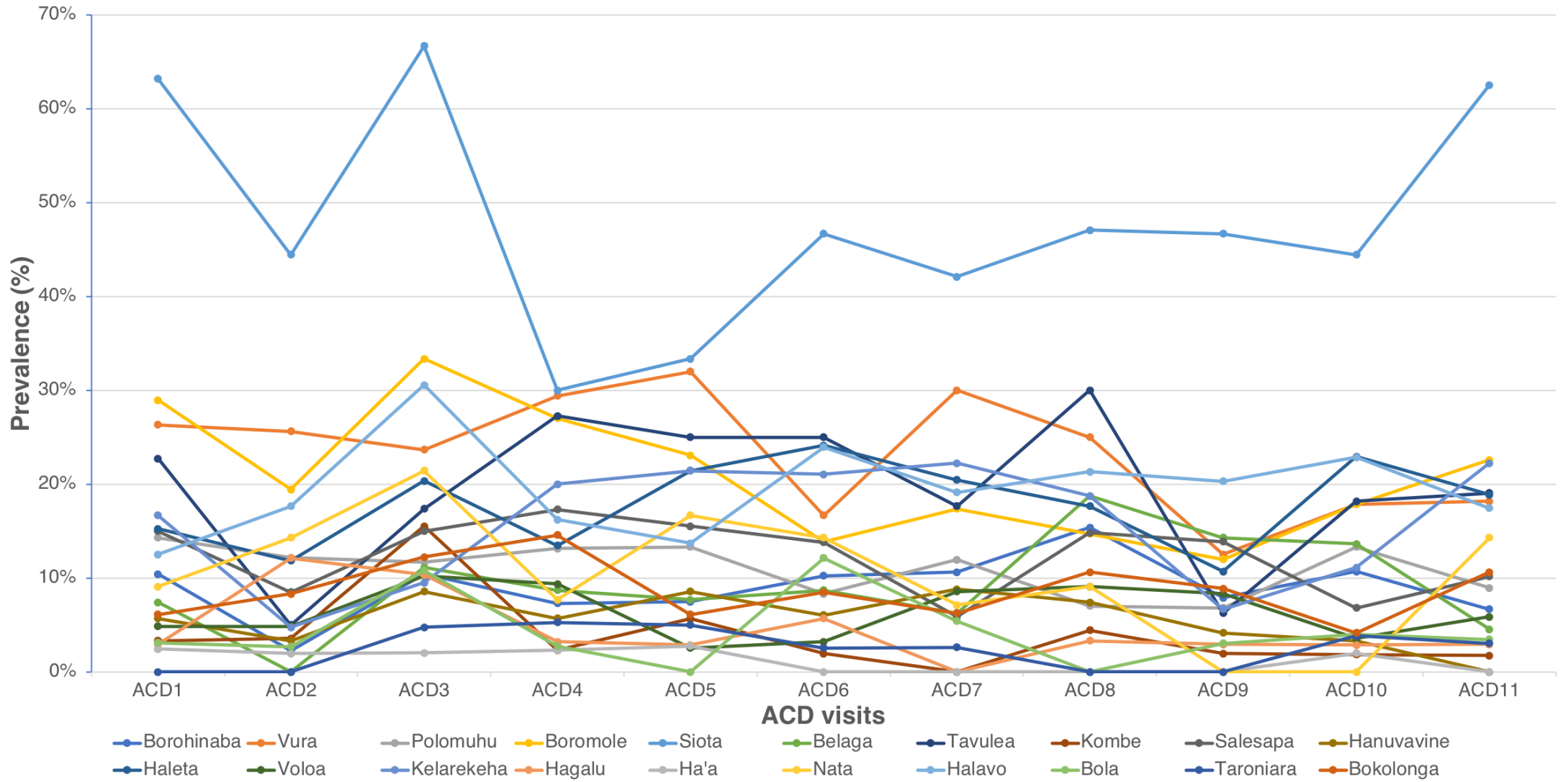

Supplement: Supplementary file 10 — Additional file 10: Figure S2. Prevalence of Plasmodium vivax infection in villages during ACD visits. [file 12936_2019_2727_MOESM10_ESM.pdf]
