# Supplementary material for: Molecular epidemiology of residual Plasmodium vivax transmission in a paediatric cohort in Solomon Islands
Source: Malar J. 2019 Mar 28;18:106. doi: 10.1186/s12936-019-2727-9 (PMC6437916; doi:10.1186/s12936-019-2727-9)
Supplement: Supplementary file 6 — Additional file 6: Figure S4. Plasmodium falciparum genotypes variants that were successfully characterized by msp2, TA81 and Polyα markers. [file 12936_2019_2727_MOESM6_ESM.pdf]

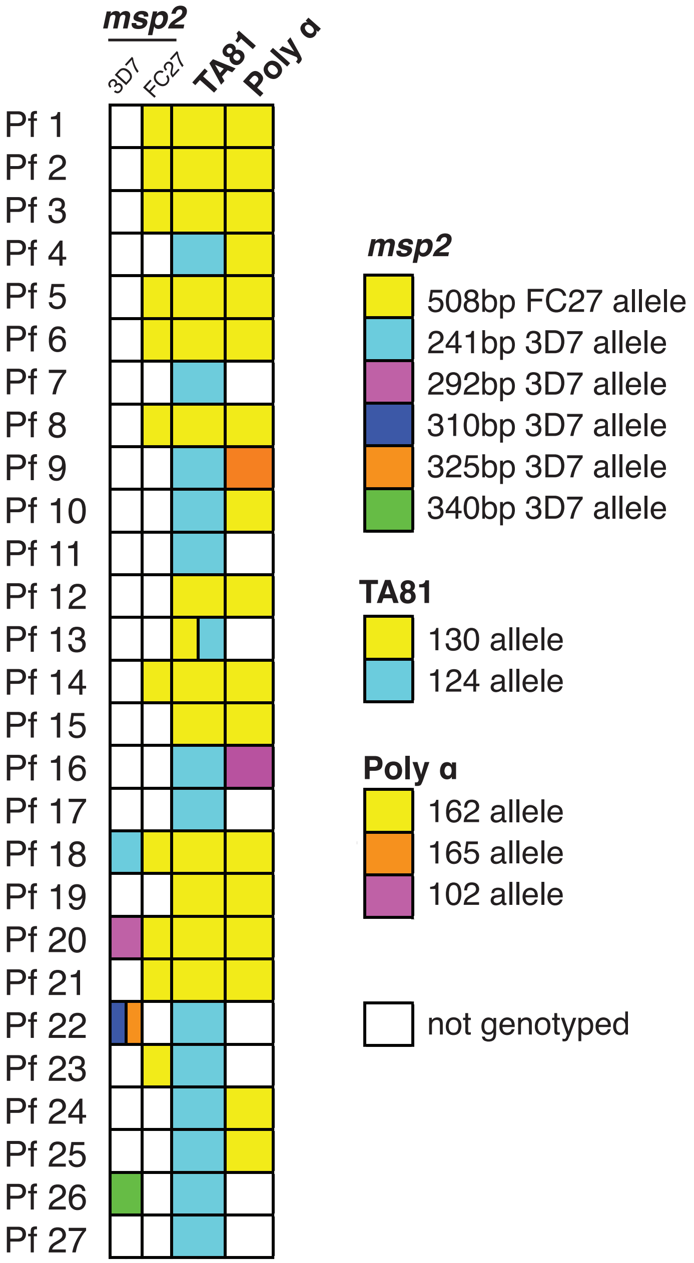

msp2
3D7
FC27
TA81
Poly α
Pf 1
Pf 2
Pf 3
Pf 4
Pf 5
Pf 6
Pf 7
Pf 8
Pf 9
Pf 10
Pf 11
Pf 12
Pf 13
Pf 14
Pf 15
Pf 16
Pf 17
Pf 18
Pf 19
Pf 20
Pf 21
Pf 22
Pf 23
Pf 24
Pf 25
Pf 26
Pf 27
msp2
508bp FC27 allele
241bp 3D7 allele
292bp 3D7 allele
310bp 3D7 allele
325bp 3D7 allele
340bp 3D7 allele
TA81
130 allele
124 allele
Poly α
162 allele
165 allele
102 allele
not genotyped
