# Supplementary material for: Molecular epidemiology of residual Plasmodium vivax transmission in a paediatric cohort in Solomon Islands
Source: Malar J. 2019 Mar 28;18:106. doi: 10.1186/s12936-019-2727-9 (PMC6437916; doi:10.1186/s12936-019-2727-9)
Supplement: Supplementary file 7 — Additional file 7: Figure S5. Asymptomatic and submicroscopic Plasmodium spp. infections during ACD1, ACD8 and ACD11 visits. [file 12936_2019_2727_MOESM7_ESM.pdf]

Plasmodium spp. infections from ACD1 (n = 824), ACD8 (n = 672) and ACD11 (n = 754)

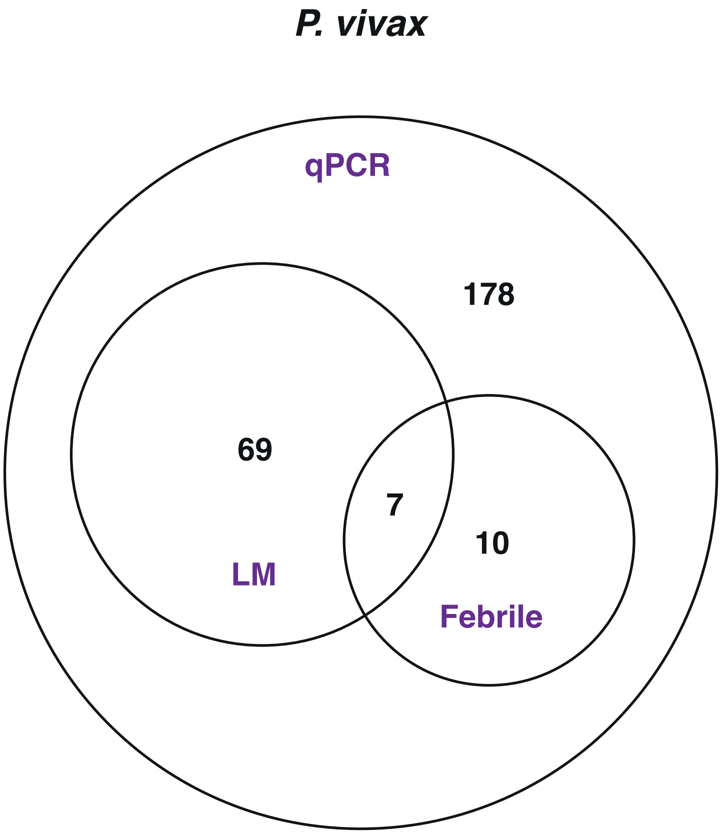

Prevalence estimates of *P. vivax* during ACD1, ACD8 and ACD11 : 11.8%

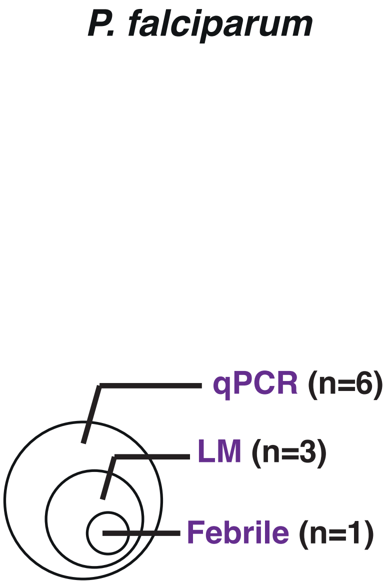

Prevalence estimates of *P. falciparum* during ACD1, ACD8 and ACD11 : 0.5%
